# Constipation

**Published:** 1881-12

**Authors:** 


### CONSTIPATION.

CONSTIPATION becomes alarmingly frequent as people acquire the means of living luxuriously without regular manual labor. It is still more frequent among persons who become so through ignorance of the penalties that follow a want of attention to the regular demands of nature.

When fecal matter is retained in the rectum more than twenty-four hours, it commences its destructive work, notably in two ways. *First*, It is absorbed into the system, and acts as a blood poison. One of the worst results from blood poisoning from constipation is to cause disease of the mind, varying from habitual moroseness to actual insanity. *Second*, By its accumulation and impacting in the lower portion of the rectum it interrupts the circulation of the blood in the adjoining parts, and may produce, from this cause alone, piles, fistula, inflammation of the bladder, disease of the prostate gland, or cancer of the rectum. In fact, most of these diseases are developed in this way. To a very great extent we hold our health and happiness, even our lives, in our own hands. If we sacrifice health and life through our ignorance, or indifference, we pay the penalty through suffering, more severe because more prolonged than instant destruction. From whatever cause, there is, unfortunately, great ignorance in regard to the laws of health among a class of people who are otherwise intelligent. How many parents are there who look carefully to the habits of their children, upon which all that will make life desirable for them so much depends? Cathartics and clysters should not be used in constipation. They increase the trouble.

That which has, perhaps, met with most general favor relates to the diet. Certain foods have been regarded as specifics for this complaint, and particularly "Graham bread." This often induces activity of the bowels, but it ruins digestion like other cathartics, in a manner which we have heretofore fully explained in the pages of this journal. Kneading the bowels has sometimes afforded relief, but it affords no permanent benefit, as a rule. We have, however, found a remedy for constipation and for piles which is always safe, and is attended with no inconvenience or discomfort. We have prescribed it in hundreds of cases. It has usually afforded prompt relief, and many patients who have suffered for years believe that they have been permanently cured of constipation by its use. This remedy is "Nelaton's Suppository." It is prepared and sold by Hall & Ruckel, wholesale druggists, 218 Greenwich street, New York, at 50 cents and $1.00 a box, and is sent free by mail on receipt of price.

